# Bedaquiline for multidrug-resistant TB in paediatric patients

**DOI:** 10.5588/ijtld.21.0022

**Published:** 2021-09-01

**Authors:** R. Moodliar, V. Aksenova, M. V. G. Frias, J. van de Logt, S. Rossenu, E. Birmingham, S. Zhuo, G. Mao, N. Lounis, C. Kambili, N. Bakare

**Affiliations:** 1 Tuberculosis and HIV Investigative Network, King Dinuzulu Hospital, Sydenham, Durban, South Africa; 2 National Medical Research Center for Phthisiopulmonology and Infectious Diseases, Moscow, Russian Federation; 3 De La Salle Health Sciences Institute, Dasmariñas City, Cavite, the Philippines; 4 Janssen Research & Development, Leiden, The Netherlands; 5 Janssen Pharmaceutica, Beerse, Belgium; 6 Janssen Research & Development, Raritan, NJ; 7 Janssen Research & Development, Titusville, NJ; 8 IQVIA, NC; 9 Johnson & Johnson Global Public Health, New Brunswick, NJ, USA

**Keywords:** BDQ/TMC207, MDR-TB, children/adolescents, pharmacokinetics, safety/tolerability

## Abstract

**BACKGROUND:**

TMC207-C211 (NCT02354014) is a Phase 2, open-label, multicentre, single-arm study to evaluate pharmacokinetics, safety/tolerability, antimycobacterial activity and dose selection of bedaquiline (BDQ) in children (birth to <18 years) with multidrug-resistant-TB (MDR-TB).

**METHODS:**

Patients received 24 weeks’ BDQ with an anti-MDR-TB background regimen (BR), followed by 96 weeks of safety follow-up. Results of the primary analysis are presented based on data up to 24 weeks for Cohort 1 (≥12–<18 years; approved adult tablet at the adult dosage) and Cohort 2 (≥5–<12 years; age-appropriate 20 mg tablet at half the adult dosage).

**RESULTS:**

Both cohorts had 15 patients, of whom respectively 53% and 40% of Cohort 1 and Cohort 2 children had confirmed/probable pulmonary MDR-TB. Most patients completed 24 weeks’ BDQ/BR treatment (Cohort 1: 93%; Cohort 2: 67%). Geometric mean BDQ area under the curve 168h values of 119,000 ng.h/mL (Cohort 1) and 118,000 ng.h/mL (Cohort 2) at Week 12 were within 60–140% (86,200–201,000 ng.h/mL) of adult target values. Few adverse event (AE) related discontinuations or serious AEs, andnoQTcF >460 ms during BDQ/BR treatment or deaths occurred. Of MGIT-evaluable patients, 6/8 (75%) Cohort 1 and 3/3 (100%) Cohort 2 culture converted.

**CONCLUSION:**

In children and adolescents aged ≥5–<18 years with MDR-TB, including pre-extensively drug-resistant-TB (pre-XDR-TB) or XDR-TB, 24 weeks of BDQ provided a comparable pharmacokinetic and safety profile to adults.

## Abstract (FR)

**CONTEXTE:**

TMC207-C211 (NCT02354014) est une étude de Phase 2, ouverte, multicentrique, à bras unique visant à évaluer la pharmacocinétique, l’innocuité/la tolérance, l’activité antimycobactérienne et à sélectionner la dose requise de bédaquiline (BDQ) chez l’enfant (de la naissance à <18 ans) atteint de TB multirésistante (MDR-TB).

**MÉTHODES:**

Les patients ont reçu 24 semaines de BDQ avec un traitement de fond (BR) de la MDR-TB, puis ont fait l’objet d’un suivi de l’innocuité pendant 96 semaines. Les résultats de l’analyse primaire sont présentés en prenant appui sur les données allant jusqu’ à 24 semaines pour la Cohorte 1 (≥12–<18 ans ; comprimé adulte homologué au dosage adulte) et pour la Cohorte 2 (≥5–<12 ans ; comprimé de 20 mg adapté à l’âge, moitiédu dosage adulte).

**RÉSULTATS:**

Les deux cohortes comprenaient 15 patients, parmi lesquels respectivement 53% et 40% des enfants des Cohorte 1 et Cohorte 2 étaient atteints de MDR-TB pulmonaire confirmée/probable. La plupart des patients ont mené à bien les 24 semaines du traitement BDQ/BR (Cohorte 1 : 93% ; Cohorte 2 : 67%). Les valeurs moyennes géométriques de l’aire sous la courbe_168h_ de la BDQ de 119 000 ng.h/mL (Cohorte 1) et de 118 000 ng.h/mL (Cohorte 2) à la Semaine 12 étaient entre 60% et 140% (86 200–201 000 ng.h/mL) des valeurs cibles adultes. Peu d’arrêts de traitement liés à des évènements indésirables (AE) ou d’AE graves ont été observés, et aucun QTcF >460 ms sous traitement BDQ/BR ni aucun décès n’ont été enregistrés. Parmi les patients évaluables par MGIT™, 6/8 (75%) de la Cohorte 1 et 3/3 (100%) de la Cohorte 2 ont été associés à une conversion de culture.

**CONCLUSION:**

Chez l’enfant et l’adolescent âgés ≥5-<18 ans atteints de MDR-TB, dont ceux atteints de TB pré-ultra-résistante (pré-XDR-TB) ou de XDR-TB, 24 semaines de BDQ ont été associées à un profil pharmacocinétique et d’innocuité comparable à celui de l’adulte.

Surveillance data on multidrug-resistant TB (MDR-TB) in children are limited.^1^ MDR-TB is a growing public health concern for children in countries with significant transmission of drug-resistant *Mycobacterium tuberculosis* strains.^2^–^5^ Worldwide, ~25,000–32,000 children develop MDR-TB each year, of whom <5% receive treatment;^3^,^6^ 5% have extensively drug-resistant TB (XDR-TB).^4^ The true burden of MDR-TB is likely to be higher because of difficulties with diagnosis and microbiological confirmation in young children.^7^,^8^ Given the unmet need for new treatments, and that dosing recommendations for existing therapies in children are often inadequate,^9^ there is a clear requirement to involve children in development programmes for new TB treatments.^10^

Bedaquiline (BDQ; TMC207), a diarylquinoline antimycobacterial,^11^ was granted accelerated or conditional approval in various countries for MDR-TB based on Phase 2 clinical data in adults;^12^–^15^ BDQ is recommended in MDR-TB treatment regimens in the WHO treatment guidelines.^16^ BDQ achieved high culture conversion and treatment success rates in adult MDR-TB and XDR-TB patients in real-world settings,^17^–^20^ and is positively associated with treatment success and reduced mortality.^21^,^22^

The primary objective of the present study is to evaluate the pharmacokinetics (PK), safety and tolerability of BDQ, combined with an anti-MDR-TB background regimen (BR) over 24 weeks in children and adolescents with pulmonary MDR-TB to support regulatory approval of BDQ for paediatric use and provide guidance on BDQ dose selection for each of the following four age cohorts (1: ≥12–<18 years; 2: ≥5–<12 years; 3: ≥2–<5 years; 4: birth–<2 years). Secondary objectives include BDQ anti-mycobacterial activity, treatment outcome, treatment adherence and palatability. The primary analysis for Cohorts 1 and 2 are presented when all patients had completed 24 weeks of BDQ treatment or had discontinued earlier.

## METHODS

### Study design

TMC207-C211 (NCT02354014) is an ongoing, Phase 2, open-label, multicentre, single-arm study in ≥60 children and adolescents aged from birth to <18 years with confirmed or probable (defined in Table 1 legend) pulmonary MDR-TB, including pre-extensively drug-resistant TB (pre-XDR-TB) or XDR-TB, who will initiate or have already begun MDR-TB treatment (Supplementary Data).

**Table 1:** Baseline patient demographics and disease characteristics

Demographic characteristics	ITT population	

	Cohort 1 (*n* = 15) *n* (%)	Cohort 2 (*n* = 15) *n* (%)
Female	12 (80)	9 (60)
Age, years, median (range)	16 (14–17)	7 (5–10)
Race		
Asian	2 (13.3)	1 (6.7)
Black	8 (53.3)	9 (60)
White	5 (33.3)	5 (33.3)
Country		
The Philippines	2 (13.3)	1 (6.7)
Russia	5 (33.3)	5 (33.3)
South Africa	8 (53.3)	9 (60)
Height, cm, median (range)	157.2 (150 175)	119 (112–147)
Weight, kg, median (range)	46.2 (38.4–75.0)	22.6 (13.9–35.5)
Body mass index, kg/m^2^, median (range)	17.9 (15.6 27.9)	16.2 (11.1–18.8)
Disease characteristics		

Cavitary disease of ≥2 cm	3 (20)	3 (20)
Baseline albumin		
High	3 (20)	2 (13.3)
Normal	12 (80)	13 (86.7)
Extent of TB-resistant strains		
Confirmed MDR-TB	11 (73.3)	10 (66.7)
Limited to INH and RIF	6 (40)	5 (33.3)
RIF-monoresistant	4 (26.7)	5 (33.3)
XDR-TB^*^	1 (6.7)	0
Probable MDR-TB^†^	4 (26.7)	3 (20.0)
Limited to INH and RIF	2 (13.3)	1 (6.7)
Pre-XDR-TB^‡^ SLI-resistant	2 (13.3)	2 (13.3)
Drug-susceptible TB^§^	0	1 (6.7)
Other^¶^	0	1 (6.7)
Previous use of second-line TB drugs	15 (100)	14 (93.3)

^*^ Defined as resistance to INH and RIF and additional resistance to any SLI (amikacin, kanamycin and capreomycin) and any fluoroquinolone.

^†^ Defined as clinical evidence of TB disease (i.e., at least one of the following signs or symptoms: persistent cough, weight loss, or failure to thrive; persistent unexplained fever; persistent unexplained lethargy or reduced playfulness; or the presence of any of the following in the neonate: pneumonia, unexplained hepatosplenomegaly, or sepsis-like illness) and immunological evidence of TB infection (i.e., a positive IGRA test result at screening if no positive IGRA test result was available within 2 months before screening) and documented exposure to a source case with MDR-TB based on a standardised questionnaire.

^‡^ Defined as resistance to INH and RIF and additional resistance to either any SLI drug (amikacin, kanamycin and capreomycin; SLI-resistant) or any fluoroquinolone.

^§^ One patient in Cohort 2 was enrolled in the study as having probable MDR-TB and treated, but was later found as having drug-susceptible TB (based on Xpert result from Day 1 that became available post-baseline), and, thus, no longer qualified for the study.

^¶^ One patient was enrolled in the study as having probable MDR-TB and treated but was found to be culture-positive for non-TB mycobacteria (results became available post-baseline) and, thus, no longer qualified for the study.

ITT = intent-to-treat; MDR-TB = multidrug-resistant TB; INH = isoniazid; RIF = rifampicin; XDR-TB = extensively drug-resistant TB; SLI = second-line injectable; IGRA = interferon-gamma release assay.

In Cohort 1, adolescent patients received BDQ adult tablets (100 mg/tablet) with a meal for 24 weeks (400 mg once daily [qd] for 2 weeks, then 200 mg thrice a week [tiw] with intakes at least 48 h apart for 22 weeks). In Cohort 2, patients received an age-appropriate oral 20 mg BDQ tablet formulation (Supplementary Data) at 200 mg qd for 2 weeks, then 100 mg tiw for 22 weeks. The short or long anti-MDR-TB BR was selected by the investigator as per WHO treatment guidelines,^23^,^24^ National Tuberculosis Programme (NTP) treatment guidelines and the current standard of care at each of the three study sites in The Philippines, Russia and South Africa (Supplementary Data). The BR included ≥3 drugs to which the *M. tuberculosis* isolate was susceptible in vitro or ≥4 drugs to which the *M. tuberculosis* isolate would likely be susceptible (in absence of in vitro testing results) (Supplementary Data). BDQ and BR administration was supervised using directly observed therapy (DOT) according to the country’s NTP guidelines or study-specific DOT instructions (as a minimum).

Patients were followed for 120 weeks post-baseline. Patients who prematurely discontinued were followed up for survival until 120 weeks post-baseline, unless they withdrew from the study.

The study protocol/amendments were reviewed by an Independent Ethics Committee/Institutional Review Board at each of the three study sites in The Philippines, Russia and South Africa. An independent data monitoring committee reviewed safety data regularly. The study was conducted in accordance with the principles of the Declaration of Helsinki and Good Clinical Practice. All patients/representatives provided written informed consent.

### Study assessments

During treatment, assessments occurred at baseline, Day 1 and Weeks 2, 4, 6, 8, 12, 16, 20 and 24 (both cohorts); additional assessments to monitor hepatic safety in Cohort 2 occurred at Weeks 1, 3, 5, 10, 14, 18, 22. Follow-up continued for 96 weeks, with visits at Weeks 28, 32, 40 and 48, then every 12 weeks until Week 120.

Adverse events (AEs) were recorded at each visit. Haematology, biochemistry and urinalysis, a urine pregnancy test, hepatitis A, B and C tests, chest X-rays, 12-lead electrocardiograms, vital signs, physical examinations, alcohol use, visual acuity testing and audiology testing (for patients on an injectable BR drug) were performed at defined visits. Blood samples were collected for BDQ and *N*-monodesmethyl metabolite (M2) plasma analyses (Supplementary Data).

Microbiological status was measured as per local standard of care using GeneXpert (Cepheid, Sunnyvale, CA, USA), acid-fast bacilli sputum smear and qualitative culturing with the mycobacteria growth indicator tube (MGIT™960™; BD, Franklin Lakes, NJ, USA) system. Drug susceptibility testing (DST) of *M. tuberculosis* isolates against BDQ and anti-MDR-TB drugs was determined at baseline (or screening) and only repeated on the last positive sample, and in case of reversion to positive culture (Supplementary Data). TB signs and symptoms (e.g., fever, cough) were assessed by the investigator for resolution (including radiological improvement).

Palatability of the paediatric BDQ formulation in Cohort 2 was assessed at Weeks 2, 4 and 24 using a visual analogue scale (VAS) with five hedonic faces completed by the child or the parent/caregiver. Adherence to study medication was assessed using a study-specific DOT card.

### Pharmacokinetic analyses

PK parameters were derived using non-compartmental analysis (Supplementary Data). Individual model-based estimation of area under the plasma concentration-time curve from the time of dose administration up to 168 hours post-dose (AUC_168h_; weekly AUC during the maintenance phase) was derived at Week 12 (primary PK endpoint) and Week 24, and compared with 60–140% of the adult geometric mean AUC_168h_ from previous studies (Supplementary Data).

### Statistical analyses

Sample size was based on the PK data and the detection of safety events (Supplementary Data). Fifteen patients were enrolled per cohort, assuming 20% of patient drop out before Week 12.

Safety analysis was performed on the intent-to-treat (ITT) population, i.e., all patients who had at least one intake of BDQ. Microbiology analyses were performed on the modified ITT population (mITT), i.e., all ITT patients with confirmed or probable MDR-TB.

As this is an open-label, single-arm study, no formal hypothesis testing was conducted. Data were summarised using descriptive statistics (number of observations and percentages, mean, standard deviation (SD), median and range, as appropriate).

## RESULTS

### Patient baseline demographics, disease characteristics and disposition

The study began on 4 May 2016. Week 24 interim analysis database cut-offs were 14 November 2017 (Cohort 1) and 10 January 2019 (Cohort 2). Overall, 21 patients were screened in Cohort 1 and 17 in Cohort 2, and 15 were enrolled in each cohort and included in the ITT populations.

Most patients were from South Africa (Cohort 1: 53.3%; Cohort 2: 60%), female (Cohort 1: 80%; Cohort 2: 60%), Black (Cohort 1: 53.3%; Cohort 2: 60%) and with confirmed/probable pulmonary MDR-TB limited to isoniazid plus rifampicin resistance (Cohort 1: 53.3%; Cohort 2: 40%) (Table 1). Median ages were 16 (Cohort 1) and 7 years (Cohort 2).

All patients in Cohort 1 and most in Cohort 2 (93.3%) had previous exposure to second-line TB drugs within 8 weeks of baseline, and no lung cavitation or cavitation <2 cm at baseline (80% in each cohort). The most frequently used BR drugs were levofloxacin (100%, both cohorts) and pyrazinamide (86.7%, both cohorts) (Supplementary Table S1).

By Week 24, 14/15 patients (93.3%; Cohort 1) and 10/15 patients (66.7%; Cohort 2) had completed BDQ treatment and were ongoing in the study (Table 2).

**Table 2:** Drug termination and exposure

Disposition	ITT population	

	Cohort 1 (*n* = 15) *n* (%)	Cohort 2 (*n* = 15) *n* (%)
Completed BDQ treatment	14 (93.3)	10 (66.7)
Total number of discontinuations	1^*^ (6.7)	5 (33.3)
Discontinuations due to AEs	—	3^†^ (20.0)
XDR-TB infection	1^*^ (6.7)	—
Non-TB mycobacteria infection	—	1^‡^ (6.7)
Drug-susceptible TB infection	—	1^§^ (6.7)
Treatment duration, weeks		
BDQ treatment duration, days, median (range)	23.9 (20–25)	23.9 (0.9–24.1)
Background regimen treatment duration, days, median (range)	42 (20–78)	61 (1.6–92.3)

^*^ One patient was identified as having XDR-TB, defined as resistance to isoniazid and rifampicin and additional resistance to any second-line injectable drug (amikacin, kanamycin and capreomycin) and any fluoroquinolone, during the treatment phase and discontinued the trial on Day 138 due to the need to be treated with disallowed medication, clofazimine.

^†^ Three AEs of hepatotoxicity (one serious considered not related to BDQ and two non-serious considered possibly related to BDQ) were aminotransferase elevations without concurrent bilirubin elevations, and no clinical signs of hepatotoxicity, and were reversible after discontinuation of BDQ and adaptations in the background regimen.

^‡^ One patient discontinued on Day 64 due to non-TB mycobacteria.

^§^ One patient discontinued on Day 6 due to drug-susceptible TB.

ITT = intent-to-treat; BDQ = bedaquiline; AE = adverse event; XDR-TB = extensively drug-resistant TB.

### Palatability and adherence

According to the palatability VAS for the 20 mg paediatric tablet (assessed at all time-points), 12/14 (86%) Cohort 2 patients indicated ‘like it very much’ or ‘like it a little’. One patient indicated ‘like it very much’ at Week 24 after a negative reaction in Week 2.

During the BDQ loading phase (Days 1–14), nearly all patients (14/15 in both cohorts, 93.3%/cohort) were 100% adherent, as measured by a study-specific DOT card (Table 3). The majority (Cohort 1: 13/15, 86.7%; Cohort 2: 10/14, 71.4%) were ≥95% adherent during the continuation phase (Day 15 up to last visit) (Table 3).

**Table 3:** Adherence to bedaquiline

Adherence	ITT population	
	Cohort 1 (*n* = 15) *n* (%)	Cohort 2 (*n* = 15) *n* (%)
Day 1–14 (loading phase)	15	15
100%	14 (93.3)	14 (93.3)
≥80–<95%	1^*^ (6.7)	0
≥0–≤50%	0	1^†^ (6.7)
Day 15 to last visit (continuation phase)	*n* = 15	*n* = 14^†^
>100–≤105%	2^*‡^ (13.3)	2 (14.3)
100%	7 (46.7)	3 (21.4)
≥95–<100%	4 (26.7)	5 (35.7)
≥80–<95%	2 (13.3)	0
≥0–≤50%	0	4 (28.6)^§^

^*^ One patient (6.7%) who was between 80% and 95% adherent in the loading phase, took two extra bedaquiline loading doses on Days 15 and 16, so had 100% compliance in the continuation phase.

^†^ One patient was discontinued during the loading phase due to rifampicin susceptibility, thus, did not enter the continuation phase.

^‡^ One patient (6.7%) took the continuation dose beyond Day 168, so also had >100% compliance during the continuation phase.

^§^ One patient was discontinued during the continuation phase due to infection with non-TB mycobacteria and three patients due to an AE of hepatotoxicity.

ITT = intent-to-treat; AE = adverse event.

### Pharmacokinetics

PK of BDQ and M2 in each cohort were in the range of those previously observed in adults (Table 4). Geometric mean BDQ AUC_168h_ in Cohort 1 was 119,000 ng.h/mL at Week 12 (*n* = 15) and 134,000 ng.h/mL at Week 24 (*n* = 14), and 118,000 ng.h/mL at Week 12 (*n* = 10) and 124,000 ng.h/mL at Week 24 (*n* = 10) in Cohort 2. These values were well within the range of 86,200–201,000 ng.h/mL, which is 60–140% of the geometric mean AUC_168h_ in adult patients dosed at 400 mg qd for 14 days, followed by 200 mg tiw (Supplementary Data).

**Table 4:** Non-compartmental pharmacokinetics of BDQ and M2 during 24 weeks of BDQ plus background regimen treatment

Pharmacokinetic parameter	Week 2 (*n* = 6)^*^ mean ± SD	Week 12 (*n* = 15) mean ± SD	Week 24 (*n* = 12) mean ± SD
ITT population Cohort 1 (*n* = 15)			
BDQ			
AUC_0–24h_, ng.h/mL	39,100 ± 32,600	26,300 ± 10,300	ND
C_min_, ng/mL	1,220 ± 1,010	544 ± 263	774 ± 420
C_max_, ng/mL	2,310 ± 1,770	1,800 ± 736	ND
T_max_, h, median (range)	2 (2–8.25)	4 (2–8)	ND
M2^§^			
AUC_0–24h_, ng.h/mL	11,700 ± 4,830	5,620 ± 1,580	ND
C_min_, ng/mL	406 ± 172	188 ± 62	256 ± 137
C_max_, ng/mL	574 ± 247	297 ± 133	ND
T_max_, h, median (range)	0 (0–8)	24 (0–24)	ND
ITT population Cohort 2 (*n* = 15)	(*n* = 13)^†^	(*n* = 10)^‡^	(*n* = 10)^‡^
BDQ			
AUC_0–24h_, ng.h/mL	60,800 ± 27,400	32,200 ± 16,300	ND
C_min_, ng/mL	1,000 ± 644	461 ± 173	626 ± 274
C_max_, ng/mL	4,560 ± 1,920	2,430 ± 1,670	ND
T_max_, h, median (range)	4 (2–8)	4 (2–8)	ND
M2^§^			
AUC_0–24h_, ng.h/mL	10,600 ± 3,250	5,400 ± 2,110	ND
C_min_, ng/mL	339 ± 142	175 ± 71	190 ± 61
C_max_, ng/mL	535 ± 180	282 ± 84	ND
T_max_, h, median (range)	6 (0–8)	8 (0–24)	ND

^*^ At Week 2, rich pharmacokinetic data were collected following the final loading dose in some patients, and after the first maintenance dose in other patients. Descriptive statistics are presented only for patients with pharmacokinetic data obtained after the final loading dose.

^†^ Two patients discontinued BDQ prior to Week 2 rich pharmacokinetic sampling: one patient due to an AE of hepatotoxicity and one patient due to drug-susceptible TB infection.

^‡^ Of the five patients excluded from the analysis, three discontinued BDQ due to an AE (hepatotoxicity), one due to drug-susceptible TB infection and one due to non-TB mycobacteria infection.

^§^ M2 is an active metabolite of BDQ with 3–6-fold lower activity than BDQ (Lui K, et al. Bedaquiline metabolism: enzymes and novel metabolites. Drug Metab Dispos 2014; 42: 863–866).

BDQ = bedaquiline; ITT = intent-to-treat; SD = standard deviation; AUC_0–24h_ = area under the plasma concentration-time curve from the time of dose up to 24 hours post dose; ND = not determined; C_min_ = minimum plasma concentration; C_max_ = maximum plasma concentration; T_max_ = time to reach C_max_;AE = adverse event.

### Safety

During 24 weeks of BDQ + BR treatment, the most frequent AEs (≥20%) regardless of cause or severity were arthralgia, acne and prolonged prothrombin time in Cohort 1 and increased blood creatinine phosphokinase, prolonged prothrombin time and hepatotoxicity in Cohort 2 (Table 5).

**Table 5:** Safety analysis during 24 weeks of BDQ + BR treatment and during the overall treatment phases

AE^*^	ITT population			

	Cohort 1 (*n* = 15)		Cohort 2 (*n* = 15)	
	
	Over 24 weeks of BDQ + BR treatment *n* (%)	During the overall treatment phase^†^ *n* (%)	Over 24 weeks of BDQ + BR treatment *n* (%)	During the overall treatment phase^†^ *n* (%)
Any AE (regardless of cause or severity)	14 (93.3)	14 (93.3)	12 (80)	12 (80)
Most common (≥20% in either cohort) AEs regardless of cause or severity^‡^				
Arthralgia	6 (40)	6 (40)	0	1 (6.7)
Acne	4 (26.7)	4 (26.7)	NA	NA
Blood creatinine phosphokinase increased	1 (6.7)	1 (6.7)	5 (33.3)	5 (33.3)
Hepatotoxicity	0	0	3^§^ (20)	3^§^ (20)
Prolonged prothrombin time	3 (20.0)	3 (20.0)	5 (33.3)	6 (40)
Any AE leading to discontinuation of BDQ	0	0	3^§^ (20)	3^§^ (20)
Any AE at least possibly related to BDQ	1 (6.7)	1 (6.7)	3 (20)	3 (20)
Hepatotoxicity	0	0	2^§^ (13.3)	2^§^ (13.3)
Nausea	1 (6.7)	1 (6.7)	NA	NA
Vomiting	1 (6.7)	1 (6.7)	1 (6.7)	1 (6.7)
Any serious AE	2^¶#^ (13.3)	2^¶#^ (13.3)	1^§¶^ (6.7)	2^§¶**^(13.3)
Any Grade 3 or 4 AE	4^¶††^ (26.7)	4^¶††^ (26.7)	8^‡‡^ (53.3)	8^‡‡^ (53.3)

^*^ AEs were coded using the Medical Dictionary for Regulatory Activities. AEs and laboratory abnormalities were graded according to the US Division of Microbiology and Infectious Diseases paediatric toxicity tables (National Institute of Allergy and Infectious Diseases. Division of Microbiology and Infectious Diseases (DMID) adult toxicity table. Bethesda, MD, USA: DMID, 2007. https://www.niaid.nih.gov/sites/default/files/dmidadulttox.pdf).

^†^ The overall treatment phase, which comprised the BDQ+ BR treatment phase and the available follow-up at the respective times of database lock was median 42 (range 20–78) weeks in Cohort 1 and 61 (range 1.6–92.3) weeks in Cohort 2.

^‡^ During the overall treatment phase, AEs that occurred in two Cohort 1 patients (13.3%) were blurred vision, eye pain, eye pruritus, hypoacusis, nausea, rash, tinnitus, URTI and vulvovaginal candidiasis, and in two Cohort 2 patients (13.3%) were acarodermatitis, otitis media, tinea faciei, upper respiratory tract infection, increased ALT, increased AST, abnormal behaviour and dry skin.

^§^ Three AEs of hepatotoxicity were one serious Grade 4 AE on Day 14 considered not related to BDQ, and two non-serious Grade 3/4 AEs considered possibly related to BDQ. All AEs were aminotransferase elevations without concurrent bilirubin elevations, and no clinical signs of hepatotoxicity; all were reversible after discontinuation of BDQ and adaptations in the BR.

^¶^ Not considered at least possibly related to BDQ by the investigator.

^#^ One patient overdosed on para-aminosalicylic acid on Day 35, lasting for 3 days. One patient had Grade 4 increased ALT, increased AST and increased blood bilirubin, all beginning on Day 83 and lasting for 28 days. After the serious AEs resolved, a modified BR (without prothionamide) and BDQ was restarted and well tolerated until the end of treatment.

^**^ Another patient experienced two Grade 3 serious AEs of aminotransferase increases 110 days after completion of BDQ treatment that were considered not related to BDQ and resulted in interruption of the BR. Aminotransferase elevations resolved and occurred without concurrent bilirubin elevations, and without clinical signs of hepatotoxicity.

^††^ One patient had Grade 4 increased ALT, increased AST and increased blood bilirubin, all beginning on Day 83 and lasting for 28 days (serious AE) and after the serious AEs resolved a modified BR (without prothionamide) and BDQ was restarted and well tolerated until the end of treatment, one patient had Grade 3 increased blood creatine phosphokinase and prolonged prothrombin time and two patients had Grade 3 prolonged prothrombin time; in all three patients, prolonged prothrombin time was not associated with any clinical signs of bleeding.

^‡‡^ Three patients had Grade 3/4 hepatotoxicity, two patients had Grade 3 increased blood creatine phosphokinase and prolonged prothrombin time, one had Grade 3 prolonged prothrombin time, one patient had Grade 3 increases in aminotransferases (without concurrent bilirubin elevations) and blood creatine phosphokinase and Grade 3 prolonged prothrombin time and one patient had increased blood creatine phosphokinase. For the four patients with Grade 3 increased blood creatinine phosphokinase, these were all ≤7x ULN, corresponding to a US DAIDS toxicity grading of Grade 2 at most and with no ECG- or muscle-related AEs. For the four patients with prolonged prothrombin time, this was not associated with any clinical signs of bleeding.

BDQ = bedaquiline; BR = background regimen; ITT = intent-to-treat; AE = adverse event; URTI = upper respiratory tract infection; ALT =alanine aminotransferase; AST =aspartate aminotransferase; ULN = upper limit of normal; DAIDS = Division of AIDS; ECG = electrocardiogram.

Most AEs were Grade 1 or 2 in severity (Table 5). No deaths occurred. Two patients in Cohort 1 and one patient in Cohort 2 reported ≥1 serious AE during BDQ + BR treatment, none of which were considered at least possibly related to BDQ by the investigator (Table 5).

During BDQ + BR treatment, hepatotoxicity-related Grade 3 or 4 AEs were observed in one Cohort 1 patient and three Cohort 2 patients. In Cohort 1, one patient had Grade 4 increases in alanine aminotransferase, aspartate aminotransferase and blood bilirubin on Day 83 and lasting for 28 days, all considered serious AEs (Table 5). After the serious AEs resolved, a modified BR (without prothionamide) and BDQ was restarted and was well tolerated until the end of treatment. In Cohort 1, no patients discontinued BDQ permanently due to an AE.

In Cohort 2, three patients discontinued BDQ permanently due to AEs of hepatotoxicity, i.e., aminotransferase elevations which reversed after BDQ discontinuation and adaptions in the BR (Tables 2 and 5). One patient discontinued due to a serious Grade 4 AE (hepatotoxicity) on Day 14, considered not related to BDQ. Two patients discontinued due to non-serious Grade 3/4 AEs of hepatotoxicity considered possibly related to BDQ. In addition, another Cohort 2 patient experienced two Grade 3 serious AEs of aminotransferase increases 110 days after completion of BDQ treatment that were considered not related to BDQ and resulted in interruption of the BR. In Cohort 2, all four cases with treatment-emergent aminotransferase elevations resolved and occurred without concurrent bilirubin elevations, and without clinical signs of hepatotoxicity.

No patients had a QTcF prolongation >460 ms or a treatment-emergent increase from baseline in QTcF interval >60 ms, although increases between 30 and 60 ms were observed in six (40%) patients in both cohorts during BDQ + BR treatment. No events of ventricular arrhythmia or torsade de pointes were reported. In both cohorts, mean bodyweight increased during BDQ + BR treatment (Supplementary Data).

Most treatment-emergent laboratory abnormalities were Grade 1 or 2. No clinically significant urine-related abnormalities were observed.

### Antimycobacterial activity

In Cohorts 1 and 2, respectively, 6/8 (75.0%) and all three (100%) MGIT-evaluable patients with confirmed MDR-TB had confirmed sputum culture conversion at Week 24 (Table 6). Seven patients in Cohort 1 and six in Cohort 2 had favourable treatment outcome at Week 24 (defined in the Supplementary Data); all were from South Africa (Table 7, Supplementary Tables S2 and S3).

**Table 6:** Confirmed sputum culture conversion at Week 24 in the mITT population (primary missing = failure)*

	24 weeks of BDQ + BR treatment mITT population	
	Cohort 1 (*n* = 15)	Cohort 2 (*n* = 13)
Patients with confirmed MDR-TB	11	10
MGIT-evaluable patients^†^	8	3
Response, *n* (%)	6 (75)	3 (100)
Non-response, *n* (%)	2 (25)	0
Discontinued with microbiological status ‘converted’, *n* (%)	0	0
Discontinued with microbiological status ‘not converted’, *n* (%)	1^‡^ (12.5)	0
Failure to convert	1^§^ (12.5)	0
Revert to positive	0	0
Re-infection	0	0

^*^ Confirmed sputum culture conversion was defined as two consecutive MGIT-negative cultures from sputum samples at least 25 days apart, with the last culture within the analysis window, no intermediate positive cultures and not followed by confirmed positive cultures. In the primary Missing = Failure (M=F) analysis, data for patients dropping out before 24 weeks were censored at the last sputum culture assessment, irrespective of culture status at the time of study drop out. Such patients were considered to have had no response.

^†^ Only MGIT-evaluable patients are included in the analysis. MGIT-evaluable patients were those diagnosed with confirmed MDR-TB at screening with a positive culture at baseline (or at screening if baseline data were missing or contaminated) and at least one post-baseline result.

^‡^ Patient discontinued at Week 20 with microbiological status ‘not converted’, as all MGIT-culture results were positive from screening to discontinuation.

^§^ Patient had negative MGIT result at Week 4; all subsequent cultures until Week 24 were reported as contaminated. Patient was, therefore, considered as ‘not converted’ per protocol definition.

mITT = modified intent-to-treat; BDQ = bedaquiline; BR = background regimen; MDR-TB = multidrug-resistant TB; MGIT = Mycobacteria Growth Indicator Tube.

**Table 7:** Favourable treatment outcome rate at Week 24 in the mITT population (primary Missing = Failure)

Outcome	24 weeks of BDQ +BR treatment mITT population	

	Cohort 1 (*n* = 15) *n* (%)	Cohort 2 (*n* = 13) *n* (%)
Favourable treatment outcome	7 (46.7) 95% CI^*^ 22.3–72.6	6 (46.2) 95% CI^*^ 20.4–73.9
No favourable treatment outcome	8 (53.3)	7 (53.8)
Evaluable confirmed TB patient does not meet microbiology criteria	2 (13.3)	0
Not completed overall prescribed TB treatment	1 (6.7)	3 (23.1)^†‡^
Global TB assessment not completely resolved^§^	7^¶^ (46.7)	5 (38.5)^‡^

^*^ From Wilson score interval with continuity correction.

^†^ The three patients who did not have a favourable treatment outcome prematurely discontinued BDQ treatment due to AE (hepatotoxicity).

^‡^ One patient had an investigator’s global assessment of ‘not resolved’ in addition to not completing the overall prescribed TB treatment.

^§^ The investigator’s global TB assessment, performed according to the Consensus Statement (Seddon JA, et al. Consensus statement on research definitions for drug-resistant tuberculosis in children. J Pediatric Infect Dis Soc 2013; 2: 100–109) is a clinical assessment of the patient’s condition that includes an assessment of signs and symptoms of TB and an evaluation of radiological improvement using the standardised criteria of the Consensus Statement. An investigator’s global TB assessment of not completely resolved means ‘partially resolved’ or ‘not resolved’.

^¶^ Note that further investigation suggested variations among investigators in their rating of signs and symptom resolution based on radiological evidence.

mITT = modified intent-to-treat; BDQ = bedaquiline; BR = background regimen; CI = confidence interval; AE = adverse event.

Up to Week 24, two Cohort 1 patients and one Cohort 2 patient had post-baseline DST data (Supplementary Data). No treatment-emergent resistance was observed for the tested BR drugs.

## DISCUSSION

The Week 24 primary analysis results of the first two cohorts from our trial comprising children aged ≥5–<12 years receiving the age-appropriate 20 mg BDQ formulation at half the adult dosing regimen and adolescents aged ≥12–<18 years receiving the adult BDQ formulation and dosing regimen showed that BDQ can be expected to provide comparable benefit to that observed in adults.

Several challenges exist for children and adolescents with MDR-TB, including difficulty in diagnosis and limited access to the new drugs BDQ and delamanid,^25^ and to the repurposed drugs clofazimine and linezolid due to lack of data on appropriate dosing to ensure safety and efficacy.^26^–^28^ Since BDQ became available, some groups, faced with limited options, have used BDQ in paediatric MDR-TB patients in the absence of supportive data, and showed good treatment responses, with no discontinuations due to AEs.^28^ Combining BDQ with other drugs, such as delamanid (although not allowed in the present study), could reduce the need for second-line injectable drugs, which are associated with irreversible toxicity, in particular hearing loss.^29^ The use of these injectable drugs was deprioritised in recent WHO guidelines.^16^

We address the lack of PK data, and assessed the safety and 24-week treatment outcomes of BDQ in adolescents and children with MDR-TB, including pre-XDR-TB and XDR-TB. Particular attention was paid to treatment adherence, which is crucial in order to make inferences based on PK data. Both adolescents and children demonstrated a very high level of treatment adherence in the BDQ loading and continuation phases.

The population PK data showed that BDQ exposures were within the range of those previously observed in adults. In Cohort 1, mean C_min_ (1,220 ng/mL ± SD 1,010), C_max_ (2,310 ng/mL ± SD 1,770) and AUC_24h_ (39,100 ng.h/mL ± SD 32,600) for BDQ at 2 weeks, and in Cohort 2, C_min_ (ng/mL 1,000 ± SD 644), C_max_ (ng/mL 4,560 ± SD 1,920) and AUC_24h_ (60,800 ng.h/mL ± SD 27,400) were comparable to those for BDQ at 2 weeks in adults with MDR-TB: C_min_ (727.9 ng/mL ± SD 256.6 [*n* = 30]), C_max_ (2,763 ng/mL ± SD 1,185 [*n* = 29]) and AUC_24h_ (32,960 ng.h/mL ± SD 12,720 [*n* = 26]).^14^ Furthermore, individual model-based predictions of Week 12 and 24 BDQ pharmacokinetic parameters in adolescents and children in our study were within 60–140% of the geometric mean AUC_168h_ obtained in adults (86,200–201,000 ng.h/mL).

The BDQ-containing treatment regimens were generally well tolerated in this younger patient population, with no new BDQ safety findings compared to adults. Arthralgia, the most commonly reported AE in adolescents (Cohort 1), and Grade 3 or 4 hepatic aminotransferase elevations observed in both cohorts, were also seen in Phase 2b BDQ studies in adults.^12^–^15^ Three Grade 3/4 AEs of hepatotoxicity (aminotransferase elevations) led to permanent BDQ discontinuation in Cohort 2, of which two were considered non-serious, but possibly related to BDQ, and one was considered serious but not related to BDQ. Patients were managed by close monitoring and further adjustments to drugs with potential hepatotoxicity in the BR. In Cohort 2 during 24 weeks of BDQ + BR treatment, no other Grade 3 or 4 AEs were considered serious or led to BDQ discontinuation, none of the serious AEs or other Grade 3/4 AEs were considered related to BDQ, and no patients died.

In both cohorts, only small treatment-emergent increases from baseline in QTcF (30–60 ms) and no absolute QTcF >460 ms were observed during 24 weeks of BDQ + BR treatment. Clofazimine was disallowed for Cohort 1 patients and permitted in Cohort 2.

While the present study was not designed to provide confirmatory evidence of efficacy, our current findings support the effectiveness of BDQ-containing regimens, as observed previously in adults,^14^,^15^ children and adolescents.^28^ In both cohorts, all patients with investigator-reported favourable treatment outcomes were from South Africa, and none were from Russia and the Philippines. Observed differences in treatment outcome could be because signs and symptoms, including chest X-ray abnormalities, had not resolved within 24 weeks and before completion of BR treatment, and/or due to variation among investigators in their rating of signs and symptom resolution (including radiological improvement).

A strength of the trial is the inclusion of difficult-to-treat patients. Limitations are the small size of the study and lack of comparator arm or HIV-positive patients, although in the next cohorts in this trial, HIV-positive patients may be enrolled. An IMPAACT (International Maternal Pediatric Adolescent AIDS Clinical Trial) network study is also evaluating the pharmacokinetics and safety of BDQ for treating MDR-TB in HIV-infected and non-HIV-infected children and adolescents (NCT02906007).

It is hoped that these data and the BDQ paediatric formulation will address the treatment gap for children with MDR-TB. With the availability of PK data for TB drugs, children can be treated more rationally, and are therefore more likely to benefit from the new developments emerging in the treatment of MDR-TB.
